# Correction: Synthesis, crystal structure and DFT studies of a methylphenyl piperazinyl dithiocarbamato zinc(ii) precursor for zinc sulfide nanophotocatalysts used for the degradation of trypan blue and rhodamine 6G dyes

**DOI:** 10.1039/d6ra90059c

**Published:** 2026-06-04

**Authors:** Fartisincha P. Andrew, Thandi B. Mbuyazi, Tshephiso R. Papo, Krishnan Rajeshwar, Peter A. Ajibade

**Affiliations:** a School of Chemistry and Physics, University of KwaZulu-Natal Private Bag X01, Scottsville Pietermaritzburg South Africa ajibadep@ukzn.ac.za; b Department of Chemistry & Biochemistry, The University of Texas at Arlington Arlington Texas 76109 USA

## Abstract

Correction for ‘Synthesis, crystal structure and DFT studies of a methylphenyl piperazinyl dithiocarbamato zinc(ii) precursor for zinc sulfide nanophotocatalysts used for the degradation of trypan blue and rhodamine 6G dyes’ by Peter A. Ajibade *et al.*, *RSC Adv.*, 2026, **16**, 1848–1862, https://doi.org/10.1039/D5RA04748J.

The University of KwaZulu-Natal has confirmed the order of the authorship of this article should be updated and the correct order is shown above.

The Royal Society of Chemistry apologises for these errors and any consequent inconvenience to authors and readers.

